# Mixed Adenoneuroendocrine carcinoma of uterine cervix: a case series and review of literature

**DOI:** 10.1093/omcr/omaf281

**Published:** 2026-01-25

**Authors:** Sara Salehiazar, Komeil Mirzaei Baboli, Elliott Lebby, Laron McPhaul

**Affiliations:** Pathology and Laboratory Medicine, Harbor-UCLA Medical Center, 1000 W Carson St, Torrance, CA 90502, United States; Pathology and Laboratory Medicine, Harbor-UCLA Medical Center, 1000 W Carson St, Torrance, CA 90502, United States; Radiology Department, Harbor-UCLA Medical Center, 1000 W Carson St, Torrance, CA 90502, United States; Pathology and Laboratory Medicine, Harbor-UCLA Medical Center, 1000 W Carson St, Torrance, CA 90502, United States

**Keywords:** GYN pathology, sexual and reproductive health, oncology, radiology

## Introduction

Cervical carcinoma is a leading cause of cancer-related mortality in women, with squamous cell carcinoma being the most common histological type and adenocarcinoma accounting for 10–25% of cases [1]. Neuroendocrine carcinoma (NEC) of the cervix is a rare malignancy, representing approximately 2% of cervical cancers [2]. The College of American Pathologists classifies cervical neuroendocrine tumors into four categories: large cell, small cell, classical carcinoid, and atypical carcinoid, with small cell neuroendocrine carcinoma being the most prevalent. This tumor type is associated with early nodal and distant metastasis, resulting in a generally poor prognosis [2–5].

Mixed Adenoneuroendocrine Carcinoma (MANEC) of the cervix is an even rarer subtype with an unfavorable prognosis, accounting for less than 1% of cervical malignancies. According to the World Health Organization (WHO), MANEC is defined by the presence of both adenocarcinoma and neuroendocrine carcinoma, with each component comprising more than 30% of tumor cells [2, 3]. Early diagnosis of MANEC of the uterine cervix is crucial because this cancer is rare, highly aggressive, and often present at an advanced stage. Detecting MANEC early can significantly improve survival rates and enable more effective, less aggressive treatment options. There is no universally accepted standard chemotherapy regimen for MANEC. Treatment is tailored to the dominant tumor component, with platinum-based regimens (Cisplatin + Etoposide or Irinotecan) for neuroendocrine features and 5-FU-based regimens (5-Fluorouracil, Capecitabine, Oxaliplatin) for adenocarcinoma features. Multimodal and individualized approaches are common due to the tumor’s aggressive behavior and variable response to therapy [6, 7].

## Method and materials

The pathology database at Harbor-UCLA Medical Center was reviewed to identify cases of MANEC of the cervix diagnosed according to WHO criteria. Patient demographic data, histopathological findings, tumor characteristics, including immunohistochemical stains and treatment modalities—were collected. In situ hybridization (ISH) was performed for HR-HPV detection, and PD-L1 expression was assessed in two cases.

## Case series

**Figure 1:**
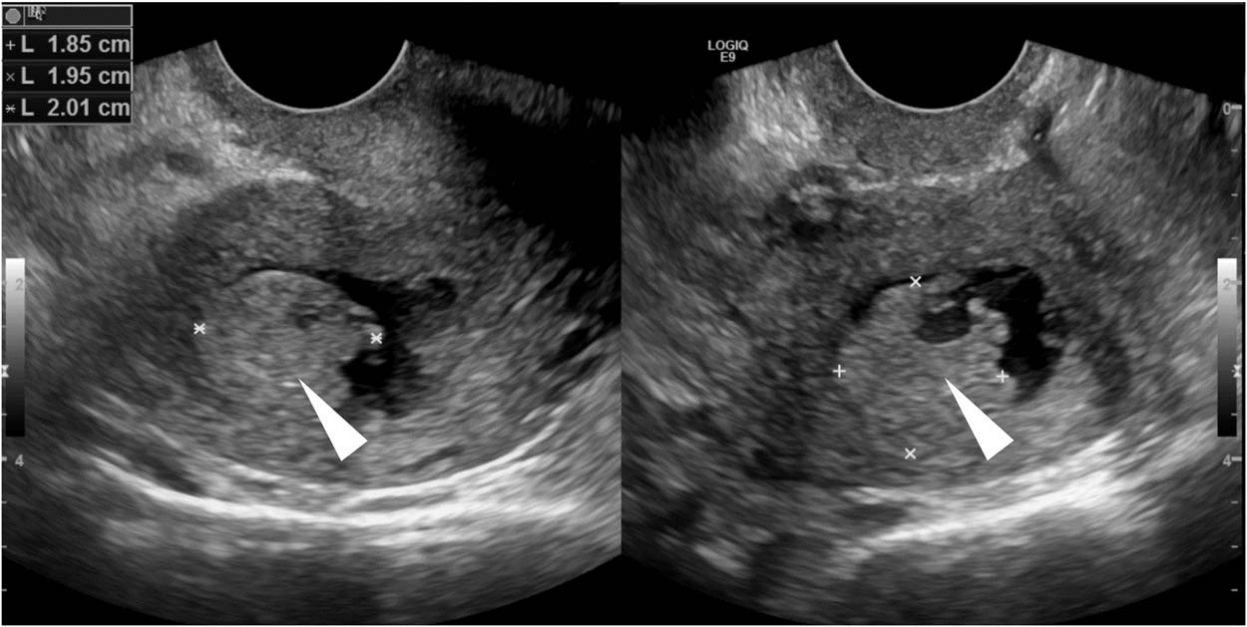
Transvaginal pelvic ultrasound demonstrates an approximately 20 mm echogenic endometrial mass (arrowhead) with irregular margins. The mass appears to invade the myometrium.

Case 1: A 57-year-old postmenopausal woman with a history of hypertension, high BMI, and goiter presented with vaginal bleeding and was initially misdiagnosed with a urinary tract infection. The initial examination was unremarkable, showing no visual cervical mass or lesions. A transvaginal pelvic ultrasound demonstrated an approximately 20 mm echogenic endometrial mass with irregular margins (Fig. 1). The mass appeared to invade the myometrium, raising concerns for malignancy.

**Figure 2:**
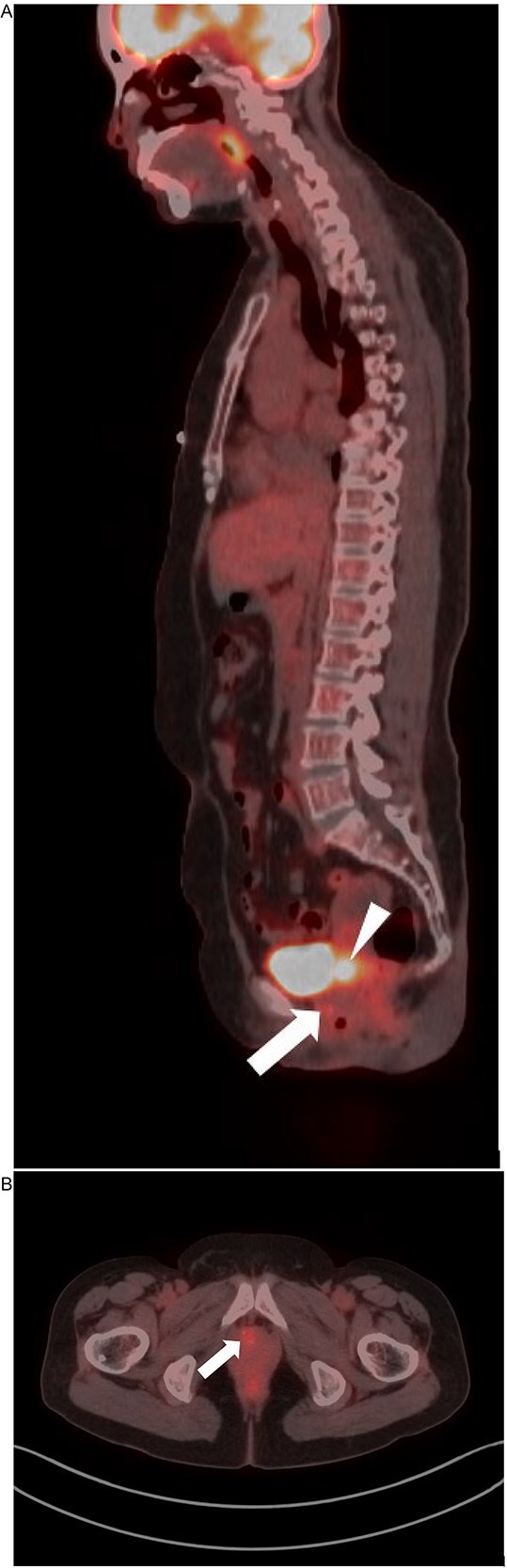
PET-CT: Sagittal (A) and axial (B) PET-CT demonstrates an approximately 2.2 cm uterine mass (arrowhead) with intense F-18 fluorodeoxyglucose (FDG) uptake, a finding that can suggest malignancy. There is an additional focus of FDG uptake in the cervix (arrow). There is normal physiologic FDG activity within the bladder.

Given the high risk of malignancy, the patient underwent a PET-CT scan from the skull base to the mid-thigh, which revealed a 2.2 cm uterine mass with intense F-18 fluorodeoxyglucose (FDG) uptake, suggesting malignancy. Additionally, there was an FDG-avid focus in the cervix (Fig. 2). A Pap smear revealed high-risk HPV positivity (HPV mRNA positive for E6/E7 from 14 high risk HPV types), and her care proceeded with cervical biopsies.

**Figure 3:**
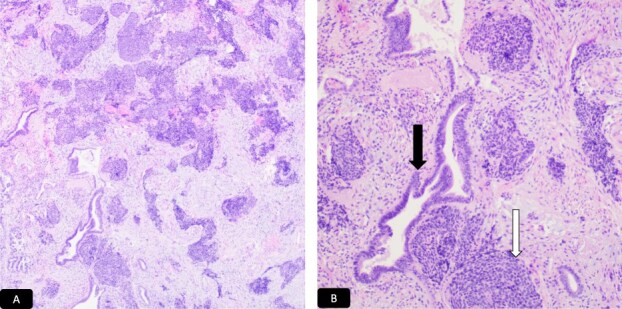
H&E microscopic picture, A-40x and B-100x, two population of malignant cells. Black solid arrow: Glandular cells and white solid arrow: Small cell population.

**Figure 4:**
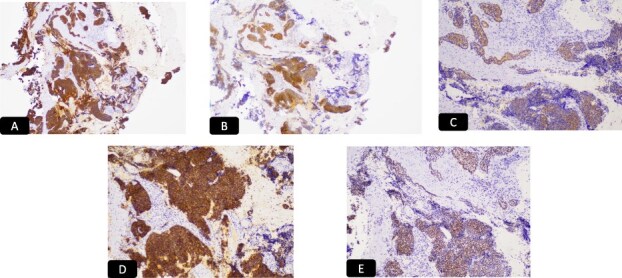
IHC stain for A: CAM 5.2, B: P 16, C: Synaptophysin, D: CD 56, E: TTF1.

Histopathological analysis of the cervical biopsies revealed two populations of malignant cells (Fig. 3A and B). The glandular and small cell components stained positive for CAM5.2 and p16 (Fig. 4A and B). The small cell component also stained positive for synaptophysin, TTF-1, and CD56 (Fig. 4C–E). Both tumor components stained negative for p40, p63, CK5/6, and chromogranin A. Notably, small cell neuroendocrine carcinoma of the cervix can stain for TTF-1. The immunophenotype and morphology were consistent with HPV-associated endocervical adenocarcinoma mixed with small cell neuroendocrine carcinoma.

The final diagnosis was HPV-associated endocervical adenocarcinoma mixed with small cell neuroendocrine carcinoma. A paraffin-embedded block was sent for PDL-1 status evaluation, which revealed a combined positive score of less than 1, indicating no PDL-1 expression. The patient was clinically staged as IIB. Due to the poor prognosis, she was not considered a good candidate for surgery and was referred for primary chemotherapy and radiation therapy.

She completed treatment with radiotherapy and cisplatin. Following therapy, a PET/CT scan revealed new innumerable pulmonary nodules throughout the lungs, the largest and most FDG-avid measuring 1.5 cm in the left upper lobe, concerning metastatic disease. There was no evidence of FDG-avid lesions within the uterus or cervix, suggesting no residual disease. Additionally, there was no evidence of regional lymphadenopathy.

The patient underwent flexible bronchoscopy with electromagnetic navigation, transbronchial biopsy, and a mediastinal lymph node survey. Cytopathological analysis of the lymph nodes showed tumor cells positive for p16, CAM5.2, TTF-1, synaptophysin, and CD56, and negative for CD45, p40, chromogranin, Napsin A, p63, and CK5/6. Ki67 demonstrated a high proliferation index (greater than 80%).

The immunophenotype and morphology of the lung lymph node are similar to the previous cervical biopsies, although the cervical biopsies also revealed an additional well-differentiated adenocarcinoma component that stained positive for p16 and negative for TTF-1, synaptophysin, and CD56. These findings are consistent with metastasis from the cervical primary tumor. As the patient did not qualify for any clinical trial, patient continued the chemotherapy regimen with Atezolizumab, carboplatin, and etoposide and after progression of disease with metastasis to lung, patient received 3 cycles of Lurbinectedin and 5 cycles of gemcitabine. She was on gemcitabine until death due to respiratory failure and pneumonia.

**Figure 5:**
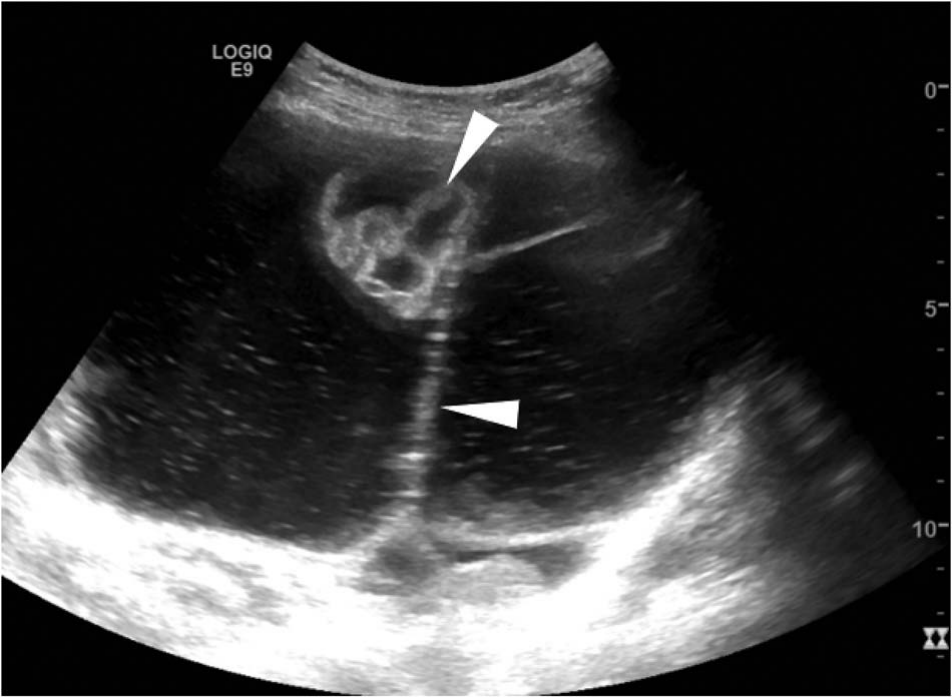
Ultrasound: Transabdominal pelvic ultrasound demonstrates an approximately 19 cm complex cystic mass occupying the lower abdomen and pelvis. There are internal solid components and septations (arrowheads), findings that are concerning for neoplasm.

**Figure 6:**
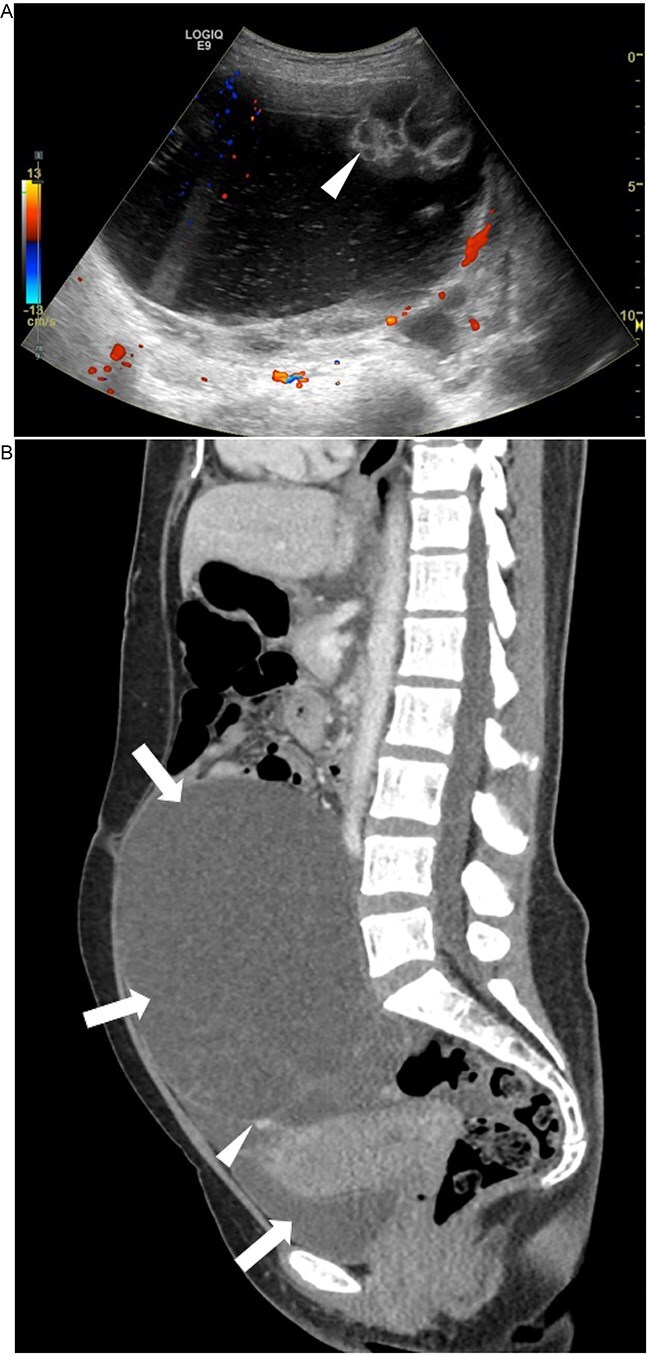
CT: Sagittal CT image demonstrates an approximately 19 cm cystic structure (arrows) occupying the lower abdomen and pelvis. There is suggestion of either complex septations and/or solid material (arrowhead).

**Figure 7:**
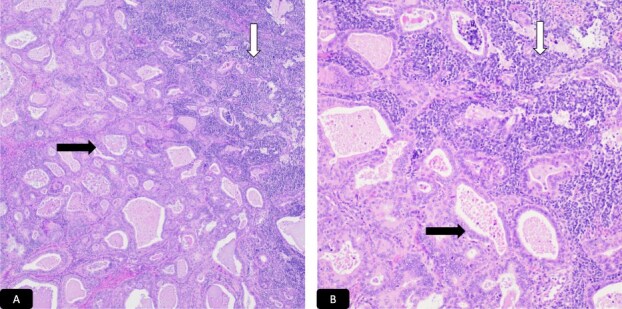
H&E microscopic picture, A-40x and B-100x, two population of malignant cells. Black solid arrow: Glandular cells and white solid arrow: Small cell population.

**Figure 8:**
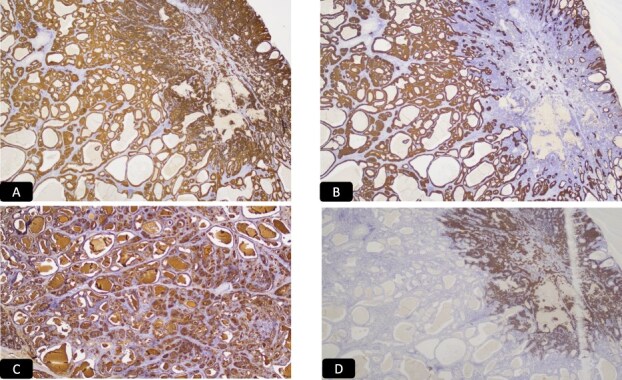
IHC stain for A: P 16, B: CAM 5.2, C: CEA D: Synaptophysin.

**Figure 9:**
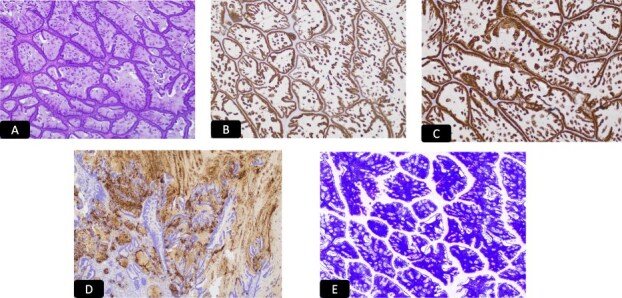
Left ovary metastasis. A: H&E, B: CK 7, C: P 16, D: CEA, E: Alcian blue/PAS.

Case 2: A 46-year-old woman with G4P3 presented with abdominal symptoms and weight loss. Transabdominal pelvic ultrasound (Fig. 5) and sagittal CT image showed an approximately 19 cm complex cystic mass occupying the lower abdomen and pelvis (Fig. 6). On examination it was revealed palpated approximately 16 cm wide and approximately 20 cm high, which is palpated to 2–3 cm above the umbilicus. Since there was a high suspicion of malignancy, the patient underwent total abdominal hysterectomy, bilateral salpingo-oophorectomy, omentectomy, and lysis of adhesion for complex pelvic mass. The adnexal mass was sent fresh for frozen evaluation; it consists of a large cystic and solid mass measuring 20 × 19 cm with a smooth surface and two small exophytic cysts. The cystic parts contain abundant mucinous material. The frozen diagnosis revealed the mucinous borderline tumor. The uterus, cervix, ovaries, and fallopian tubes were normal grossly with no lesions. Microscopically the cervix showed cervical carcinoma that resembles endocervical adenocarcinoma of usual type with a minor component with features of neuroendocrine carcinoma (Fig. 7A and B). The predominant adenocarcinoma component stains positive for P16 and CAM 5.2, focally positive for CEA, ER, and P40, and negative for vimentin, synaptophysin, and chromogranin (Fig. 8A–C). The minor component with neuroendocrine features stains positive for synaptophysin and p16, focally positive for CAM5.2 (Fig. 8A–D), and negative for chromogranin and vimentin. Immunophenotype and morphology are consistent with the classification adenocarcinoma admixed with neuroendocrine carcinoma. The sections from the adnexal mass with mucinous carcinoma involving the left ovary has atypical mitosis (Fig. 9A) and stains positive for CK7 and p16, focally positive for CEA, and negative for CK-20, PAX-8, and ER. Mucin stains (Alcian blue/PAS and mucicarmine) (Fig. 9B–E) demonstrate intracellular mucin. The immunophenotype and morphology are most consistent with metastasis from cervix primary adenocarcinoma.

**Figure 10:**
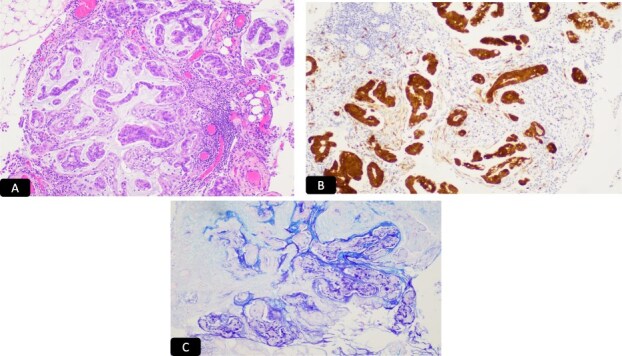
Omentum metastasis. A: H&E, B: P 16, C: Alcian blue/PAS.

Stroma associated with the right ovarian endometriotic cyst stains positive for, consistent with endometriotic cyst. The tumor involving omentum is microscopic (Fig. 10A–C).

The synoptic protocol demonstrated as pT4 as the tumor extending beyond the true pelvis.

PD-L1 CPS was 2 demonstrating PD-L1 expressions. Chemotherapy started with 4 cycles of topotecan, paclitaxel, and bevacizumab and despite treatment, follow up imaging showed metastasis to the lung and breast, chemotherapy regimen changes to 2 cycles of gemcitabine.

The biopsy of the breast revealed small cell neuroendocrine carcinoma with the similar morphology to the primary tumor, with positive synaptophysin, and weak NSE. There was no PDL1 expression showing the high grade of the nature of these metastatic tumors. The breast biopsy was sent for NGS testing and it showed NOTCH3 (67%) and STAG2 (23%) mutations with MSI stable and tumor mutational burden of 8.9 m/MB. Currently patients live in nursing facility and receive tramadol for pain control and waiting for clinical trial.

**Figure 11:**
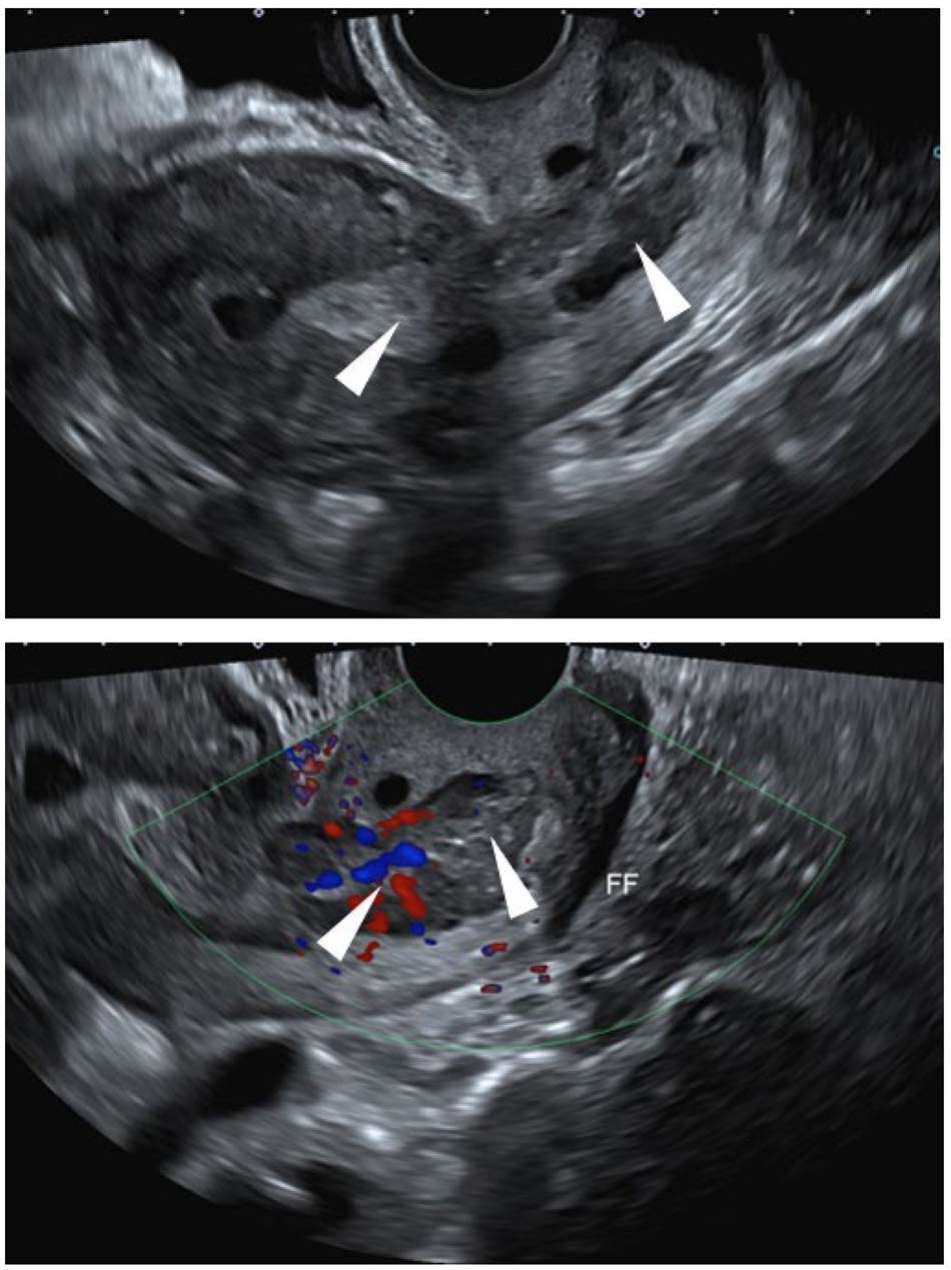
Ultrasound: Transvaginal pelvic ultrasound demonstrates thickening and heterogeneity in the lower endometrial stripe and endocervical canal (arrowheads). There is also pelvic free fluid (FF).

**Figure 12:**
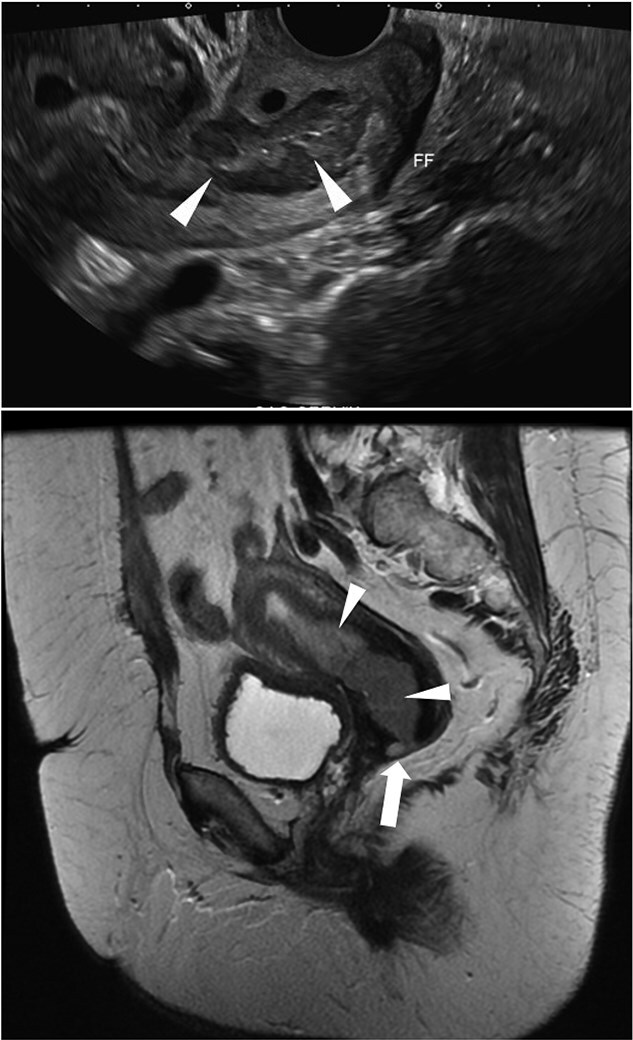
MRI: Pelvic MRI demonstrates a lobular soft tissue mass within the endometrial canal (arrowheads). The lesion extends from the superior endometrium to the level of the external os of the cervix with likely cervical stromal invasion (arrow).

**Figure 13:**
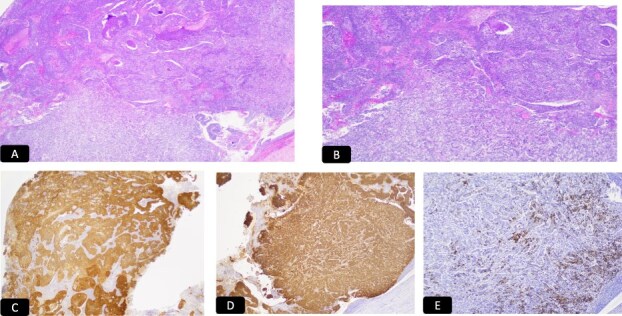
H&E microscopic picture, A-40x, B-100x, C: P 16, D: pCEA, E: Chromogranin.

Case 3: A 59-year-old postmenopausal woman presented with intermittent bleeding for eight months. Transvaginal ultrasound showed thickening and heterogeneity in the lower endometrial stripe and endocervical canal with pelvic free fluid (Fig. 11). MRI of the pelvic demonstrated a lobular soft tissue mass within endometrial canal which extends from superior endometrium to the level of the external os of the cervix (Fig. 12). Colposcopy revealed firm cervix without gross lesion. Patient underwent endometrial biopsy which revealed neoplastic proliferation with small areas of glandular structures and sheets of highly atypical cells with increase nuclear/cytoplasmic ratio and hyperchromatic nuclei. Numerous mitosis and areas of necrosis are present. IHC studies revealed tumor cells are block positive for P16, positive for CEA and ER and negative for P40, Vimentin, P63, CK5/6 and PR. P53 showed normal (wild type) presentation. HPV high risk (ISH) was positive favoring a cervical primary. Chromogranin (neuroendocrine marker) is focally positive and synaptophysin was negative. MMR study (MLH1, PMS2, MSH2 and MSH6) revealed intact nuclear expression. Initial biopsy suggested poorly differentiated adenocarcinoma mixed with neuroendocrine Carcinoma, HPV associated, most consistent with cervical primary. The patient underwent radical hysterectomy, bilateral salpingo-oophorectomy and bilateral pelvic and para-aortic lymph node dissection. The resected specimen showed 4.5 cm tan-red mass involving the lower uterine segment. The tumor is composed of a conventional cervical adenocarcinoma with villoglanduar pattern, occupying approximately 60% of the tumor and a solid the cells have pleomorphic and hyperchromatic nuclei with glandular chromatin and eosinophilic cytoplasm (Fig. 13A and B) and show patchy positive staining for chromogranin (Fig. 13C–E), consistent with a neuroendocrine phenotype. INSM1 was negative in glandular and solid part and synaptophysin also was negative in both glandular and solid part concur with the biopsy result. The proliferation index was high with Ki67 approaching 70–80%. Based on morphology and immunophenotype, the cased diagnosed as mixed non endocrine and neuroendocrine carcinoma. Following the surgery patient underwent adjuvant chemo radiation therapy given neuroendocrine component and continued surveillance in every 3 months. Imaging follow-up showed no metastasis.

## Discussion

Adenocarcinoma is a type of invasive epithelial tumor characterized by glandular differentiation, featuring atypical cells, hyperchromatic nuclei, and pleomorphism. The confirmation is reinforced by the presence of mitotic figures and infiltration into the cervical stroma. Adenocarcinoma constitutes approximately 20% of all cervical cancers. While cervical screening programs effectively decrease the occurrence of invasive squamous cell carcinoma of the cervix, their efficacy in identifying precursor lesions of adenocarcinoma is comparatively lower [2]. Neuroendocrine carcinomas are infrequent, making up approximately 2%–5% of all cervical carcinomas. Typically, affected patients are younger, with a median age of 42 years. The College of American Pathologists has proposed a new classification system for neuroendocrine tumors of the cervix, which includes large cell, small cell, classical carcinoid, and atypical carcinoid subtypes. Small cell neuroendocrine tumors exhibit small round cells with minimal cytoplasm, abundant mitotic figures, and extensive necrosis. Classical carcinoid tumors lack cytologic atypia, necrosis, and have rare mitotic figures. In contrast, atypical carcinoid tumors show cytologic atypia, ≤ 10 mitotic figures/HPF, and focal necrosis. The histologic criteria for diagnosing cervical large cell neuroendocrine tumors involve the presence of large cells with vesicular nuclei and prominent nucleoli, a mitotic index exceeding 10/10 HPFs, and geographic areas of tumor necrosis. Additionally, these tumors feature neurosecretory granules with a minimal amount (<5%) of glandular or squamous components [2, 4].

In another classification, NEC is categorized into two groups: low-grade, encompassing typical and atypical carcinoid tumors, and high-grade, comprising small cell and large cell neuroendocrine carcinomas. Compared to other types of cervical cancer, there is a higher frequency and earlier occurrence of lymph node involvement and distant metastases. Given that aggressive surgery alone is insufficient for achieving remission, postoperative adjuvant chemotherapy and/or radiotherapy are typically required. The prognosis for Mixed Adenoneuroendocrine Carcinoma (MANEC) is akin to that of small cell NEC. Recurrences in small cell neuroendocrine Carcinoma (SCNEC) typically manifest in extra-pelvic areas such as bone, lymph nodes, supraclavicular region, and lungs. This aggressive tumor is marked by elevated recurrence rates and distant metastases, even in its early stages [2, 8].

As per the WHO classification, Mixed Adenoneuroendocrine Carcinoma (MANEC) is characterized by a blend of adenocarcinoma and neuroendocrine carcinoma, with each component constituting more than 30% of the tumor cells and the incidence is less than 1% in cervical cancer [2].

Adenocarcinoma and neuroendocrine carcinoma are more commonly linked to Human Papillomavirus type 18 than squamous cell carcinoma. Notably, a crucial diagnostic indicator is the punctate nuclear positivity of Human Papillomavirus detected through in-situ hybridization in the endocervical adenocarcinoma component of these tumors. Hence, both cytological examination and Human Papillomavirus genotyping play significant roles in the diagnostic process [2, 8–10].

In a majority of cervical Small Cell Neuroendocrine Carcinoma (SCNEC) cases, there is a notable presence of immunoreactivity for p16, signifying a high risk of HPV infection [11].

The cytological characteristics of SCNEC closely resemble those found in other organs like the lungs. The neoplastic cells exhibit uniform small size (typically < 3 times larger than small resting lymphocytes), minimal cytoplasm, finely granular chromatin (‘salt and pepper’ appearance), inconspicuous nucleoli, and nuclear molding [10, 11]. The precision of cytological diagnoses for cervical SCNEC is noted to be limited, primarily owing to the infrequency of occurrences of cervical SCNECs [11].

Differential diagnostic considerations for cervical SCNEC encompass squamous cell carcinoma, malignant lymphoma, melanoma, and sarcoma. In squamous cell carcinoma, neoplastic cells typically exhibit varying amounts of dense cytoplasm, occasionally demonstrating keratinization and a distinct cytoplasmic border. The presence of koilocytotic atypia aids in the diagnosis of squamous cell carcinoma. Also, nuclear molding is usually absent in squamous cell carcinoma Malignant lymphoma is characterized by individual neoplastic cells without clustering, lacking nuclear molding, with nuclei displaying coarse chromatin and prominent nucleoli. As previously outlined, the cytological points of differentiation for SCNEC involve the presence of nuclear molding, salt and pepper chromatin, and a necrotic background. Taking these features into consideration is crucial for arriving at an accurate diagnosis [3, 8, 10].

Immunohistochemical detection of neuroendocrine differentiation in small cell cases involves the use of markers such as chromogranin A, synaptophysin, CD56, neuron-specific enolase (NSE), and the cell proliferation index (Ki-67) [2–4]. It is important to note that in cervical neuroendocrine carcinoma the IHC marker may be negative [3].

Chromogranin A (CG-A) is an acid glycoprotein, exclusively found in dense core granules. It serves as a storage site for peptide and catecholamine hormones in endocrine organs and neuroendocrine cells. Tumor cells in individuals with liver metastases exhibit notably higher concentrations of CG-A compared to cells that metastasize to lymph nodes. The sensitivity and specificity of CG-A in such cases were estimated to be approximately 60%–100% [2].

Synaptophysin, stands out as the most specific tumor marker. Its detection is possible in endocrine cells and small synaptic membrane vesicles. Ki-67 serves as a marker for cell proliferation, correlating with histopathological parameters and tumor grading. According to the WHO, tumor grading is determined by the percentage of Ki-67 and the number of mitoses. Specifically, Grade 1 (G1) is assigned if Ki-67 is < 2% and mitosis is < 2, Grade 2 (G2) if Ki-67 is 3%–20% and mitosis is 2–20, and Grade 3 (G3) if Ki-67 is > 20% and mitosis is > 20. Based on Ki-67 and the number of mitoses, the tumor is categorized into NET (grade 1 and 2) and NEC (grade 3), representing high grade [2].

## Conclusion

Mixed Adenoneuroendocrine Carcinoma of the uterine cervix presents unique diagnostic and therapeutic challenges due to its rare occurrence and aggressive nature. Early detection of Mixed Adenoneuroendocrine Carcinoma (MANEC) of the uterine cervix is critical, as this rare and highly aggressive malignancy is often diagnosed at an advanced stage. Timely diagnosis can substantially enhance survival outcomes and allow for more effective and potentially less intensive treatment strategies. Currently, there is no universally established chemotherapy protocol for MANEC. Treatment typically targets the predominant histologic component: platinum-based regimens are used for tumors with neuroendocrine features, while 5-FU–based regimens are favored for tumors with glandular differentiation. Due to the tumor’s aggressive nature and heterogeneous response to treatment, a multimodal and individualized therapeutic approach is often required.

The case series demonstrates the variability in clinical presentation, treatment response, and outcomes among MANEC patients. The aggressive progression observed in Cases 1 and 2 highlights the limitations of current therapeutic strategies, underscoring the need for innovative treatments. Combining surgery with chemotherapy (and sometimes radiotherapy) is considered optimal, especially for early-stage disease, as it addresses both local and systemic disease and improves outcomes. For advanced or metastatic MANEC, chemoradiation or palliative chemotherapy is used to control symptoms and prolong survival. Targeted therapy is not yet standard but may be considered in select cases based on molecular profiling. Molecular testing can identify potential targets, though evidence for benefit is still emerging.

Briefly the best outcomes for MANEC of the uterine cervix are achieved with a multimodal approach: surgery for early disease, followed by platinum-based chemotherapy, and consideration of targeted therapy in select cases. This strategy improves survival, reduces recurrence, and offers the best chance for disease control in this aggressive cancer.

Future directions in the management of MANEC include a stronger focus on molecular profiling and targeted therapies. Advances in next-generation sequencing and immunotherapy hold promise for personalizing treatment approaches, potentially improving survival rates for this aggressive cancer. Collaborative efforts between researchers, oncologists, and pathologists are critical for developing comprehensive management protocols and advancing our understanding of MANEC.

Moreover, large-scale studies and clinical trials are needed to identify effective systemic therapies and explore the potential of novel agents, such as PARP inhibitors or drugs targeting NOTCH and other relevant pathways. Efforts to standardize diagnostic criteria and treatment protocols will help optimize outcomes for patients with this rare and challenging malignancy.

Ultimately, improving awareness and understanding of MANEC among healthcare professionals is crucial. A multidisciplinary approach that integrates clinical, radiologic, histopathologic, and molecular data can significantly enhance diagnostic accuracy and treatment efficacy, potentially leading to better outcomes for patients afflicted with this aggressive form of cervical cancer.
